# Imaging Dynamic Peroxynitrite Fluxes in Epileptic Brains with a Near‐Infrared Fluorescent Probe

**DOI:** 10.1002/advs.201900341

**Published:** 2019-06-11

**Authors:** Jiong‐sheng Hu, Chenwen Shao, Xueao Wang, Xiaojiao Di, Xuling Xue, Zhi Su, Jing Zhao, Hai‐Liang Zhu, Hong‐Ke Liu, Yong Qian

**Affiliations:** ^1^ School of Chemistry and Materials Science Nanjing Normal University Wenyuan Road 1 Nanjing 210046 China; ^2^ State Key Laboratory of Pharmaceutical Biotechnology School of Life Sciences Nanjing University Xianlin Road 163 Nanjing 210023 China

**Keywords:** brain, epilepsy, fluorescent probe, near‐infrared imaging, peroxynitrite

## Abstract

Epilepsy is a chronic neurodegenerative disease, and accumulating evidence suggests its pathological progression is closely associated with peroxynitrite (ONOO^−^). However, understanding the function remains challenging due to a lack of in vivo imaging probes for ONOO^−^ determination in epileptic brains. Here, the first near‐infrared imaging probe (named ONP) is presented for tracking endogenous ONOO^−^ in brains of kainate‐induced epileptic seizures with high sensitivity and selectivity. Using this probe, the dynamic changes of endogenous ONOO^−^ fluxes in epileptic brains are effectively monitored with excellent temporal and spatial resolution. In vivo visualization and in situ imaging of hippocampal regions clearly reveal that a higher concentration of ONOO^−^ in the epileptic brains associates with severe neuronal damage and epileptogenesis; curcumin administration can eliminate excessively increased ONOO^−^, further effectively protecting neuronal cells. Moreover, by combining high‐content analysis and ONP, a high‐throughput screening method for antiepileptic inhibitors is constructed, which provides a rapid imaging/screening approach for understanding epilepsy pathology and accelerating antiseizure therapeutic discovery.

Epilepsy is a chronic neurodegenerative disease characterized by recurrent unpredictable convulsions.^1^ Despite the increasing availability of antiepileptic drugs in the past two decades, more than 30% of patients are medically intractable response to them.^2^ Existing drug development for antiepileptic strategies merely provides basic symptomatic treatment, yet has not been successful in addressing the pharmacoresistant problem and preventing epileptogenesis after status epilepticus.^3^ Increasing evidence indicated that epilepsy is closely associated with oxidative stress, and significant increases of the reactive oxygen/nitrogen species (ROS/RNS) levels can be observed in the brain of epilepsy disease.^4^ In this sense, the factors of oxidative stress should be considered in further antiepileptic therapeutic strategies.^3^ Therefore, a better understanding of the dynamic neurochemistry process underlying epilepsy in living beings would be beneficial in the early diagnosis and prevention as well as the search for novel therapeutics.

Brain injury resulting from epileptic seizure is a complex dynamic process associated with excitotoxicity‐induced mitochondrial dysfunction and oxidative stress.^5^ During the initiation and progression of epilepsy, numerous ROS are continuously generated that further reacts with nitric oxide (NO) to produce RNS such as peroxynitrite (ONOO^−^),^6^ thereby resulting in an accumulation of oxidative stress by reacting with many bioactive molecules including proteins, nucleic acids, and lipids, which can further cause neuronal cell death.^3^, ^7^ The overexpressed ONOO^−^ is considered a critical neurotoxic factor that plays an important role in the pathogenesis of epilepsy, which can served as a potential biomarker to predict epilepsy early.^8^, ^9^ Nevertheless, the potential biological roles of ONOO^−^ in epileptogenesis have not yet been fully understood. Thus, to explore the pathophysiological mechanism of in vivo ONOO^−^ and investigate its role in epilepsy, it is crucial to develop effective imaging tools for monitoring ONOO^−^ in the brain.

Fluorescent imaging with activity‐based sensing probes to study biological species in living biosystems has received great attention due to their high sensitivity, selectivity, real‐time, and noninvasiveness.^10^, ^11^ Although a few fluorescent probes have been reported for ONOO^−^ imaging in cells or tissues,^12^ in vivo imaging methods for ONOO^−^ determination in brains, including epilepsy brains, are still lacking (Table S1, Supporting Information). In addition, imaging probes that can be applied for constructing a screening platform to rapidly screen antiepileptic agents are in shortage as well. To achieve these purposes, several challenges are present: 1) the major challenge of developing probes is whether the probe could effectively cross the blood–brain barrier (BBB) to achieve imaging in the brain region;^11^, ^13^ 2) the probe with near‐infrared (NIR) excitation and emission are preferred to obtain deeper tissue penetration, less photodamage, and less interference of background fluorescence;^14^ 3) high selectivity and sensitivity of probe are needed to effectively monitor ONOO^−^ in the real physiological environments.^15^

Herein, we report a NIR fluorescent probe that can effectively trace endogenous ONOO^−^ signals in kainate (KA)‐induced epileptic seizure. This probe ONP, judiciously designed with a methylene blue (MB)‐based near‐infrared fluorophore, enables efficient and selective imaging ONOO^−^ in vitro and in vivo. Importantly, it can effectively cross the BBB with the brain‐targeted characteristic. Using this probe, the dynamic changes of ONOO^−^ fluxes during therapy for KA‐induced epilepsy were first observed directly in vivo and ex vivo. Moreover, for the first time, our studies have shown that a high‐throughput screening method for antiepileptic inhibitors has been constructed by combining high‐content analysis (HCA) with ONP, which provides a simple and effective approach for investigating ONOO^−^ in biosystems and for further screening antiepileptic drugs.

To monitor ONOO^−^ activity in vivo, the choice of a suitable fluorophore is a key component of the successful design of ideal fluorescent probes that can be used in the complex biological context. Our interest is focused on the MB because of its excellent pharmacokinetic and photophysical properties.^16^ It has been approved by the Food and Drug Administration (FDA) for clinical therapy, including antidepressant, antidote, antimalaria, methemoglobinemia treatment, etc. In addition, MB has been used as an ideal imaging agent for in vivo imaging studies because of its desired absorption and emission in NIR region (>640 nm), which can significantly reduce the interference of the autofluorescence. Particularly, reduction of MB to leucomethylene blue (LMB) would break the π‐conjugation in the backbone of MB fluorophore, thereby completely eliminating its absorption and fluorescence. We, therefore, envisioned that the reduced MB scaffold would be suitable for constructing NIR‐fluorescence‐based probe for sensing specific analytes, ONOO^−^. In the design of our probe, by using MB to cross the blood‐brain barrier, a boronic ester as a selective responding moiety for ONOO^−^ was incorporated into the reduced MB scaffold to obtain the final fluorescent probe ONP (**Figure** **1**A).^17^ We hope that this free probe would exhibit a very weak absorption and emission owing to the blockage of the π‐conjugated system of the fluorophore. When ONP is attacked by ONOO^−^, the boronic ester moiety would be easily removed, and newly formed a corresponding LMB that would be further oxidized to MB, resulting in the recovery of strong fluorescence in the NIR imaging window (>690 nm), which is crucial for deep tissue imaging in vivo. These significant changes can be used for sensitive monitoring intracellular ONOO^−^ and providing dynamic information for NIR fluorescent imaging of the whole brain.

**Figure 1:**
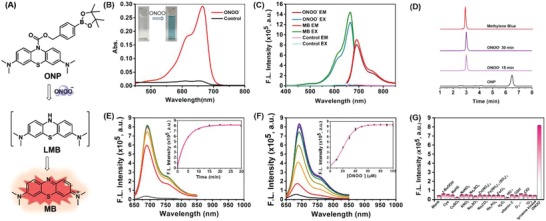
Response of ONP toward ONOO^−^. A) The proposed response mechanism of ONP. B) The absorption spectra of ONP (10 × 10^−6^ m) in the absence or presence of ONOO^−^ (100 × 10^−6^ m). C) The excitation and emission of ONP (10 × 10^−6^ m) upon addition of ONOO^−^ (100 × 10^−6^ m), MB as the standard reference. D) HPLC analysis of a reaction mixture containing ONP (10 × 10^−6^ m) and ONOO^−^ (100 × 10^−6^ m) for different incubation time in fluorescence channel (665 nm) and ONP channel (254 nm). E) Fluorescence emission spectra of ONP (10 × 10^−6^ m) after treatment with ONOO^−^ (100 × 10^−6^ m) for different incubation time (0–30 min). Inset: Time‐dependent changes in the fluorescence intensity of ONP at 692 nm. F) Fluorescence emission spectra of ONP (10 × 10^−6^ m) incubated with various concentrations of ONOO^−^ (0–100 × 10^−6^ m) for 15 min. Inset: Concentration‐dependent changes in the fluorescence intensity of ONP at 692 nm. G) Fluorescence intensity of ONP (10 × 10^−6^ m) at 692 nm in the presence of various biological species (100 × 10^−6^ m), except Vitamin C (2 × 10^−3^ m), glutathione (5 × 10^−3^ m), and tyrosine kinase (150 U mL^−1^). All data were acquired in phosphate‐buffered saline (PBS) buffer (10 × 10^−3^ m, pH 7.4, 5% MeCN) at 37 °C with excitation at 640 nm, the data represent the average of three independent experiments.

The synthetic route for ONP was shown in Scheme S1 in the Supporting Information and fully characterized by ^1^H and ^13^C NMR spectroscopy and mass spectrometry (see the Supporting Information). As expected, the ONP (10 × 10^−6^ m) alone exhibited a very weak absorbance and fluorescence, whereas provided a prominent NIR excitation (640 nm) and emission (650–850 nm). Upon incubation with ONOO^−^ (100 × 10^−6^ m) for 30 min, a remarkable absorbance band and fluorescence intensity enhancement at 665 and 692 nm were observed (Figure 1B,C). The fluorescent changes within various pHs buffers revealed that ONP was fairly stable under physiological conditions and exhibited a relatively wide range of pH applications (Figure S1, Supporting Information). High performance liquid chromatography (HPLC) analyses of the reactions indicated that the released product peak (MB) was gradually increased with a time‐dependent manner (Figure 1D). After treatment with increasing ONOO^−^ concentration, a similar tendency was observed, and the excitation/emission (Ex/Em) spectra of the final reaction mixture were also consistent with that of MB, confirming that the formation of product was indeed promoted by reacting with ONOO^−^ (Figure S2, Supporting Information, and Figure 1C). Time‐dependent fluorescence response was investigated by recording the time course of fluorescence intensity changes at 692 nm. We found that the reaction was fast, an apparent enhancement was clearly observed after 1 min incubation, which would be completed within 15 min (Figure 1E). Moreover, a concentration‐dependent fluorescence enhancement was observed when incubating ONP with increasing concentration of ONOO^−^ (0–100 × 10^−6^ m), suggesting that ONP was not only rapidly responsive to ONOO^−^, also highly sensitive toward low concentration of ONOO^−^ (Figure 1F). Remarkably, an excellent linear relationship (*R* ^2^ = 0.9979) between the fluorescent intensity at 692 nm and the concentrations of ONOO^−^ ranging from 0 to 4 × 10^−6^ m was observed (Figure S3, Supporting Information). The limit of detection (LOD) of ONP was as low as 94 × 10^−9^ m (signal‐to‐noise ratio (S/N) = 3). Furthermore, its selective responses toward various reactive species were examined, such as tert‐butyl hydroperoxide (*^t^*BuOOH), hydrogen peroxide (H_2_O_2_), hypochlorite (ClO^−^), singlet oxygen (^1^O_2_), hydroxyl radical (•OH), superoxide anion radical (O_2_^•−^), and human tyrosine kinase (150 U mL^−1^). The strongest response for ONOO^−^ and minimal fluorescence changes for other species were observed, suggesting that high specificity of ONP toward ONOO^−^ (Figure 1G). To eliminate the potential interference of common metal ions, anions, and ROS species, the selectivity of ONP toward ONOO^−^ was validated in the presence of these species mixture, suggesting that ONP has strong anti‐interference ability (Figure S4, Supporting Information). In agreement with the fluorescence “turn‐on” response, treatment of ONP with ONOO^−^could also induce a dramatic change in its UV–vis absorption profile, resulting in a remarkable enhancement of absorbance at 665 nm with a time‐dependent pattern, which can also be directly observed by the naked eye (Figures S5 and S6, Supporting Information). In addition, log *P* of ONP was 2.19 in an octanol/water system, and the quantum yield (φ) was 0.007 with MB as the reference. These results indicated that ONP is a desirable candidate for ONOO^−^ detection.

Having confirmed the efficiency of ONOO^−^‐triggered fluorescence turn‐on response, we next moved on to investigate its feasibility for dynamically mapping ONOO^−^ in live cells. Cell cytotoxicity of ONP was first evaluated by Cell Counting Kit‐8 assays, suggesting extremely low cytotoxicity of ONP to live cells (Figure S7, Supporting Information). The photostability of ONP was also examined in live cells, suggesting excellent stability of fluorescence and good performance for long‐term traceability in living cells (Figure S8, Supporting Information). The live human neuroblastoma SH‐SY5Y cells were preincubated with or without 100 × 10^−6^ m SIN‐1 (3‐morpholino‐sydnonimine, an ONOO^−^ donor) for 1 h, then treated with ONP (10 × 10^−6^ m) for another 30 min before imaging. A weak fluorescence signal was observed in cells loaded with ONP in the absence of SIN‐1; however, an important fluorescence enhancement was detected in the SIN‐1 simulated cells. In contrast, the increasing fluorescence could be efficiently suppressed by treatment with 50 × 10^−6^ m FeTMPyP, a decomposing catalyst of ONOO^−^. Pretreatment with NOC‐18 ((Z)‐1‐[N‐(2‐aminoethyl)‐N‐(2‐ammonioethyl)amino]diazen‐1‐ium‐1,2‐diolate, a NO donor, 1 × 10^−3^ m); however, no detectable change of fluorescence signal was observed compared with the control (**Figure** **2**A,B). Moreover, pretreatment with H_2_O_2_ (0.5 × 10^−3^ m) or lipopolysaccharide (LPS, 1 µg mL^−1^), a dramatic fluorescence enhancement in these cells was observed, suggesting that cellular ONOO^−^ levels increased under stimulation of external oxidative stress (Figure 2C). Interestingly, live cells that pretreated with an ROS scavenger *N*‐acetyl cysteine (NAC, 1 × 10^−3^ m), or a nitric oxide synthase inhibitor aminoguanidine (0.5 × 10^−3^ m), exhibited decreased fluorescence signals, suggesting a decreased intracellular ONOO^−^ (Figure 2D). The subcellular distribution of intracellular ONOO^−^ was investigated by costaining with ONP and different commercial organelle‐targeting agents (Figure S9, Supporting Information). We found that the fluorescence of ONP in red channel overlapped better with that of ER‐ and Mito‐Tracker Green in green channel (Pearson's coefficients were 0.90 and 0.89, respectively) over with that of lysosome (Pearson's coefficient: 0.79), suggesting that ONP has good cell‐membrane permeability, and it is an excellent fluorescent tool for imaging ONOO^−^ in live cells. These results demonstrated the feasibility of ONP for selective visualizing the dynamic changes of endogenous ONOO^−^ in live cells and revealed that the biosynthesis of cytotoxic reactive ONOO^−^ was regulated by intracellular NO and oxidative stress.

**Figure 2:**
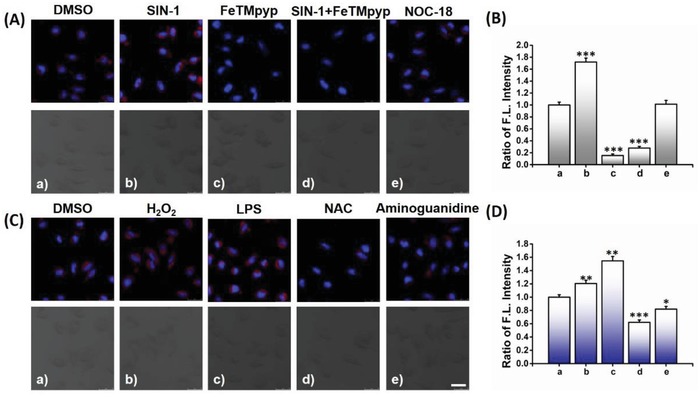
Characterization of ONP for imaging ONOO^−^ in living human neuroblastoma SH‐SY5Y cells. A) Cells were pretreated with or without SIN‐1 (100 × 10^−6^ m), FeTMpyp (50 × 10^−6^ m), and NOC‐18 (1 × 10^−3^ m) for 1 h, imaging after incubation with ONP (10 × 10^−6^ m) and Hoechst33342 (5 µg mL^−1^) for another 30 min. SIN‐1 as an exogenous ONOO^−^ donor, FeTMpyp as an ONOO^−^ decomposition catalyst, and NOC‐18 as a NO donor. Red channel: ONP; Blue channel: nucleus. Scale bars = 25 µm. B) The relative ratio of fluorescence intensity shown in (A) was quantified. C) Cells were preincubated with or without H_2_O_2_ (500 × 10^−6^ m, 1 h), LPS (1 µg mL^−1^, 12 h), NAC (1 × 10^−3^ m, 4 h), Aminoguanidine (500 × 10^−6^ m, 0.5 h), imaging after incubation with ONP (10 × 10^−6^ m) for another 30 min. Red channel: ONP; Blue channel: nucleus. Scale bars = 30 µm. D) Effects of different stimulus on changes in ONP fluorescence in (C) that were quantified. All the data represent the average of three independent experiments, the error bars were ± standard deviation (SD). Statistical analyses performed with a two‐tailed Student's *t*‐test with unequal variance, *p‐value < 0.05, **p‐value < 0.01, ***p‐value < 0.001.

To evaluate whether ONP is an effective identification tool for screening inducers and inhibitors against ONOO^−^ formation, a fluorescence‐based screening method was first constructed by combining ONP with HCA. Pretreatment of live SH‐SY5Y cells with 20 × 10^−6^ m of natural products that exhibited potential anticancer activities for 12 h, images and quantitative analyses were performed by HCA after incubation of ONP (10 × 10^−6^ m) to screen the potential inducers for promoting ONOO^−^ formation (**Figure** **3**A,B). We found that these reported anticancer agents can induce the overproduction of endogenous ONOO^−^, suggesting the oxidative stress from overaccumulation of ONOO^−^ might be one of the anticancer mechanisms.^18^ Moving forward, to investigate the screening capability of this method to identify potential inhibitors against ONOO^−^ formation, a chemical library containing different antioxidants was further built up (Figure 3C,D). The inhibitory efficiency of these compounds against ONOO^−^ formation was then screened by comparing the fluorescent intensity of ONP using HCA analysis, indicating the most of these compounds can be used to inhibit the production of ONOO^−^ except protocatechuic acid and naringenin. Importantly, curcumin, a previously reported antiepilepsy agent,^19^ was observed that can effectively control the endogenous ONOO^−^ formation. Overall, inducers and inhibitors against ONOO^−^ formation could be simply screened and identified by using high‐content analysis combined with ONP, exhibiting a variation in its NIR fluorescence signal in the presence of these agents.

**Figure 3:**
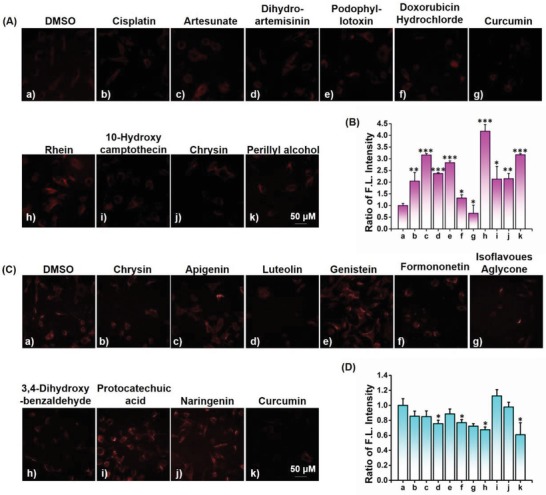
High‐content analysis for rapid imaging ONOO^−^ in living SH‐SY5Y cells. A) Cells were pretreated with various anticancer agents (20 × 10^−6^ m) for 12 h, then images were acquired after incubation with ONP (10 × 10^−6^ m) for another 30 min. B) The relative ratio of fluorescence intensity shown in (A) was quantified. C) Cells were pretreated with different antioxidants (20 × 10^−6^ m) for 12 h, then images were acquired after incubation with ONP (10 × 10^−6^ m) for another 30 min. D) The relative ratio of fluorescence intensity shown in (C) was quantified. Scale bars = 50 µm. All the data represent the average of three independent experiments, the error bars were ±SD. Statistical analyses performed with a two‐tailed Student's *t*‐test with unequal variance, *p‐value < 0.05, **p‐value < 0.01, ***p‐value < 0.001.

To investigate whether ONP can be used for mapping the dynamic changes of ONOO^−^ fluxes in vivo, a group of five‐week‐old BALB/c nude mice were performed with intraperitoneal (i.p.) injection of different agents to induce the changes of endogenous ONOO^−^. Images were captured at different time points after intravenous (i.v.) injection of ONP (Figure S10, Supporting Information). Interestingly, we found that ONP can effectively penetrate the BBB and healthy control brains have a certain concentration of ONOO^−^. Pretreatment with 3,4‐dihydroxybenzaldehyde and curcumin, the NIR fluorescence signals in brains were significantly lower than those in the control group at 5, 15, 30, 45, and 60 min, suggesting these antioxidants can effectively relieve ONOO^−^ stress in the brain. In contrast, the fluorescence signal in the SIN‐1 treated mice was mainly located at the abdominal part, likely because ONOO^−^ could be rapidly released after i.p. injection of SIN‐1. A slightly higher NIR fluorescence signal in rhein treated brains could be due to the overactivity of ONOO^−^ upon rhein treatment. These findings indicate that the endogenous ONOO^−^ levels dynamically change in brains under the exogenous stimulus can be detected by ONP.

To investigate whether ONP can be used for imaging endogenous ONOO^−^–level changes in epileptic brains, we used KA‐induced BALB/c mouse model, a widely used epilepsy mouse model.^20^ A relative level of ONOO^−^ in epileptic brains and healthy control brains were compared at the different time points. We found that the NIR fluorescence signals in KA‐induced epileptic brains were remarkably higher than those in the control group at 5, 15, 30, 45, and 60 min after i.v. injection of ONP. Moreover, after i.p. injection of KA, the concentration of ONOO^−^ in brains at 12 h exhibited a significant increase when compared with that at 24 h groups. Relative differences [*R*_(epilepsy)_/*R*_(WT)_ = *F*_(epilepsy)_/*F*_(WT)_] between epilepsy brains and wild type (WT) brains were decreased from 1.4‐fold to 1.2‐fold within 60 min (**Figure** **4**A,B), indicating that overexpressed reactive ONOO^−^ was found in the progression of epilepsy.^9^, ^21^ Importantly, pretreatment with curcumin (60 mg kg^−1^, 3 d) can successfully prevent the overproduction of ONOO^−^ that induced by KA. Meanwhile, we also found that post‐treatment of curcumin can effectively clear the overaccumulated ONOO^−^ in the KA‐induced mice compared with that of nontreatment groups, suggesting that curcumin might be an excellent antiepileptic agent for reducing the ONOO^−^ damage and preventive intervention in epilepsy. Furthermore, the findings of ex vivo NIR fluorescence images and the Z‐stack images of brain slices were also consistent with the in vivo imaging results, suggesting a higher ONOO^−^ level in epileptic brains could be effectively cleared by curcumin administration (Figure 4C and Figures S11 and S12, Supporting Information). Taken together, these results suggested that ONP imaging provided a promising approach for monitoring the dynamic changes of endogenous ONOO^−^ during therapy with antioxidant drugs in live epileptic mice.

**Figure 4:**
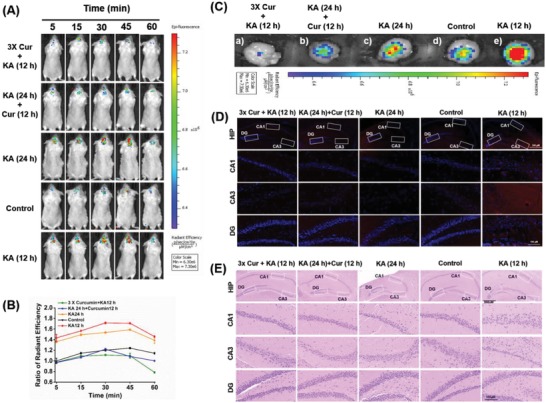
In vivo and ex vivo NIR fluorescence imaging with ONP. A) Images of healthy WT and epilepsy mice at 5, 15, 30, 45, and 60 min after intravenous injection (i.v.) of ONP. B) Quantification of images in (A). Note that NIR fluorescence signals in KA‐induced epilepsy mice were significantly higher than those in the healthy WT group. Treatment with curcumin would help to relieve the overexpressed ONOO^−^. C) Ex vivo fluorescence images of relative ONOO^−^ levels in mice brains after 60 min postinjection of ONP using the in vivo imaging system (IVIS) Spectrum imaging system. D) All ex vivo brain slices were collected after 60 min postinjection of ONP, fluorescent imaging was performed for evaluating ONOO^−^ dynamic changes and neuronal damage in the whole hippocampal region (HIP), and CA1, CA3, dentate gyrus (DG) subregions after KA administration. Red = ONP channel; Blue = 4′,6‐diamidino‐2‐phenylindole (DAPI) channel. Scale bar = 100 µm. E) HE staining for evaluating neuronal damage in the hippocampal region after KA administration. Scale bar = 100 µm.

Epilepsy is often associated with serious histological damage in the hippocampus.^22^ To determine the potential role of ONOO^−^ in KA‐induced epileptic seizure, fluorescence intensity and hippocampal neuronal death in CA1, CA3 and dentate gyrus (DG) subregions after KA exposure were investigated by ONP imaging and hematoxylin‐eosin staining (HE) staining of nearby sections (Figure 4D,E). Only weak fluorescence signal was observed in all hippocampal regions of the normal control group, accompanying with a clear hierarchical structure, neatly arranged nerve cells, intact cell membrane, and uniform cytoplasmic staining. In contrast, a significant fluorescence enhancement was clearly observed in epileptic brain slices, particularly in the CA1 and CA3 regions, which could be ascribed to the higher ONOO^−^ generation in epileptic brains (Figure S13, Supporting Information). It is worth noting that excessive ONOO^−^ produced by stimulation stress could lead to severe neuronal death, including neuronal loss and disordered arrangement in hippocampal regions, which is also consistent with previously reported dramatic reduction of the protective superoxide dismutase 2 (SOD2) and glutathione peroxidase (GPx) in epilepsy.^22^ Importantly, we found that curcumin administration can inhibit or eliminate excessively increased ONOO^−^ in all of the hippocampal subregions, further effectively protecting or reducing neuronal damage in the epileptic condition (Figure S14, Supporting Information). These observations suggested that high concentrations of ONOO^−^ may contribute to severe neuronal damage and epileptogenesis, and effective inhibition of overexpressed ONOO^−^ might be a potential treatment for epilepsy.

In summary, the results presented here have demonstrated that a novel NIR probe, ONP, could be used to track the dynamic changes of ONOO^−^ in live cell and in vivo. Importantly, the concentration of ONOO^−^ in the healthy brain is about 300 × 10^−9^ m, which would be significantly increased in epilepsy disease. Our new probe that designed basing on methylene blue, a drug approved by the FDA, can effectively cross BBB and visualize the endogenous ONOO^−^ fluxes for the first time in hippocampus of KA‐induced epileptic brains with excellent temporal and spatial resolution, revealing a positive correlation between abnormal ONOO^−^ levels and severe neuronal damage and epileptogenesis. Moreover, by combining with HCA and ONP, we successfully constructed a high‐throughput screening method for antiepileptic inhibitors by clearing overexpressed ONOO^−^. Our imaging approach could provide a highly sensitive, selective, and BBB‐permeable ONOO^−^ imaging, which could help for better understanding epilepsy pathology and accelerating the further discovery of potential antiepileptic drugs.

## Experimental Section

All mice were purchased from the Model Animal Research Centre of Nanjing University (Nanjing, China). All mice were maintained under specific pathogen‐free conditions and used in accordance with the animal experimental guidelines set by the Institute of Animal Care and Use Committee. This study has been approved by the Institutional Animal Care and Use Committee of Nanjing University and Nanjing Normal University. Further experimental details are available in the Supporting Information.

## Conflict of Interest

The authors declare no conflict of interest.

## Supporting information

Supplementary
