# Supplementary material for: Imaging Dynamic Peroxynitrite Fluxes in Epileptic Brains with a Near‐Infrared Fluorescent Probe
Source: Adv Sci (Weinh). 2019 Jun 11;6(15):1900341. doi: 10.1002/advs.201900341 (PMC6685465; doi:10.1002/advs.201900341)
Supplement: Supplementary file 1 — Supplementary [file ADVS-6-1900341-s001.pdf]

Copyright WILEY-VCH Verlag GmbH & Co. KGaA, 69469 Weinheim, Germany, 2019.

Supporting Information

for *Adv. Sci.,* DOI: 10.1002/advs.201900341

Imaging Dynamic Peroxynitrite Fluxes in Epileptic Brains with a Near-Infrared Fluorescent Probe

Jiong-sheng Hu, Chenwen Shao, Xueao Wang, Xiaojiao Di, Xuling Xue, Zhi Su, Jing Zhao, Hai-Liang Zhu, Hong-Ke Liu,* and Yong Qian**

Copyright WILEY-VCH Verlag GmbH & Co. KGaA, 69469 Weinheim, Germany, 2019.

Supporting Information

Imaging dynamic peroxynitrite fluxes in the brain of epilepsy with a near-infrared fluorescent probe

Jiong-sheng Hu,[†,a] Chenwen Shao,[†,b] Xueao Wang,[b] Xiaojiao Di,[a] Xulin Xue,[a] Zhi Su,[a] Jing Zhao,[b] Hai-Liang Zhu ,[*,b] Hong-ke Liu,[*,a] Yong Qian[*, a]

J.S. Hu, X. Di, Dr. X. Xue, Prof. Z. Su, Prof. H.K. Liu, Prof. Y. Qian

School of Chemistry and Materials Science, Nanjing Normal University, Wenyuan Road 1, Nanjing 210023, China.

Email: yongqian@njnu.edu.cn; liuhongke@njnu.edu.cn.

C. Shao, X. Wang, Prof. J. Zhao, Prof. H.L. Zhu

State Key Laboratory of Pharmaceutical Biotechnology, School of Life Sciences, Nanjing University, Xianlin Road 163, Nanjing 210023, China.

Email: yongqian@nju.edu.cn; zhuhl@nju.edu.cn.

†Both authors contributed equally to this work.

Content

1. General materials and experimental methods 3-4

2. Synthesis and characterization of ONP 4-5

3. Determination of the fluorescence quantum yield (Φ) 5

4. The limit of detection (LOD) of ONP 5-6

5. The measurement of lipophilicity 6

6. HPLC analysis 7

7. Cell culture 7

8. Cytotoxicity assay 7

9. Fluorescent imaging studies 8

10. Screening by high-content analysis 8

11. In vivo NIR fluorescence imaging with ONP 8-9

12. Frozen sectioning 9

13. Paraffin section and HE staining imaging 9-11

14. Supplemental Figures 12-24

15. NMR and MS Spectra 25

16. Reference. 30

1. General materials and experimental methods

All commercially available compounds were used as provided without further purifications. Chemicals and solvents were purchased from the companies Sigma Aldrich, Aladdin, Bepharm, *etc.* TLC analysis was performed on silica gel plates and chromatographic purification of products was performed on silica gel (300-400 mesh). ^{1}H NMR, ^{13}C NMR were recorded on a Bruker DRX400 (400 MHz), using $CDCl_3$, and DMSO-d_6 as solvents. Data are given in the following order: chemical shift (δ) values are reported in ppm with the solvent resonance as internal standard ($CDCl_3$: δ = 7.26 ppm for ^{1}H, δ = 77.16 ppm for ^{13}C; DMSO-d_6: δ = 2.50 ppm for ^{1}H, δ = 39.52 ppm for ^{13}C); multiplicities are indicated as: brs (broad singlet), s (singlet), d (doublet), dd (double doublet), t (triplet), q (quartet), m (multiplet); coupling constant values are given in Hertz (Hz). ESI-MS spectra were obtained on AGILENT 1290-6460C system (AGILENT Co., Ltd., US).

Buffer solution (PBS 1 x, pH 7.4) was prepared using deionized water. ONP was dissolved in acetonitrile (MeCN) to obtain a 1.0 mM stock solution. Hydrogen peroxide (H_2O_2), hypochlorite (ClO^-) and *tert*-butyl hydroperoxide (TBHP) were delivered from commercial aqueous solutions, respectively. Superoxide solution ($O_2{\bullet}^-$) was prepared by adding KO_2 into dry dimethyl sulfoxide (DMSO) and stirring vigorously for 10 min. Singlet oxygen ($^1O_2$) was generated in situ by addition 1 equiv. of the H_2O_2 stock solution into a solution containing 10 eq. of HClO. Hydroxyl radicals (•OH) was generated by Fenton reaction, $FeCl_2$ was added in the presence of 10 equiv. of H_2O_2. Peroxynitrite ($ONOO^-$) solution was prepared following the reported literature. Briefly, a mixture of sodium nitrite (0.6 M) and hydrogen peroxide (0.7 M) was acidified with hydrochloric acid (0.6 M), and sodium hydroxide (1.5 M) was added within 1−2 s to make the solution alkaline (ref: *Chem. Sci., 2017, 8, 4006-4011*) The concentration of peroxynitrite was estimated by using an extinction coefficient of 1670 M^{-1} cm^{-1} at 302 nm. C_{ONOO^-} = $Abs_{302\ nm}$ /1.67 (mM). The pH measurements were carried out with a PHS-25 pH meter (Shanghai INESA Co. Ltd).

UV absorption spectra were accomplished on a PerkinElmer 650 spectrophotometer (PerkinElmer Ltd., US). All fluorescence measurements were recorded at room temperature with a FLS-980 fluorescence spectrometer (Edinburgh Instruments Ltd., England) or F-4600 spectrophotometer (HITACHI, Japan). HPLC spectra were performed on an AGILENT 1200 system (AGILENT Co., Ltd., US). The imaging experiments were carried out using a confocal fluorescent microscope (Leica TCS SP8 MP, Nanjing University).

2. Synthesis and characterization of ONP

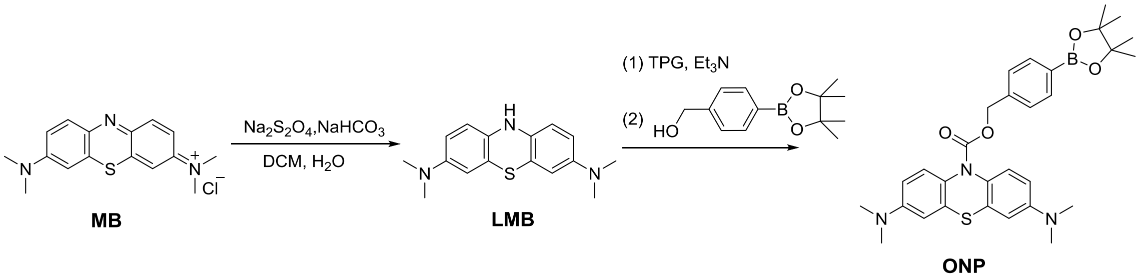

Scheme S1. The chemical synthesis route of ONP.

A mixture of methylene blue (374 mg, 1 mmol), 10 mL DCM, and 10 mL water were stirred in a 50 mL round-bottom flask under argon atmosphere. $Na_2S_2O_4$ (525 mg, 1.5 mmol) and $NaHCO_3$ (168 mg, 2 mmol) were slowly added to the stirred solution. The mixture was then stirred for 20 minutes until the aqueous phase had turned to yellow. The organic layer was separated and the aqueous layer was extracted with dichloromethane (2 X 5 mL). The organic phases were combined and dried over anhydrous sodium sulfate. Under Ar_2, the dried organic phases were quickly poured into a round-bottom flask containing triethylamine (TEA, 170 μL, 1.2 mmol). Triphosgene (TPG, 120 mg, 0.32 mmol) in 1 mL DCM was slowly added into the reaction mixtures. Upon completion of dropwise addition, the reaction was stirred at room temperature for another 0.5 h. 4-Hydroxymethylphenylboronic acid (234 mg, 1.0 mmol) and triethylamine (140 μL, 1.0 mmol) were added to the solution. After stirring overnight, the solvent was evaporated and the crude material was extracted with ethyl acetate (3 X 20 mL)

and washed with water. The crude product was purified by fast column chromatography (silica gel/chloroform-ethyl acetate) and recrystallization from acetonitrile yielding the product as a yellow solid (53.8 mg, 10%). ^{1}H NMR (400 MHz, DMSO-d_6) δ 7.91 (d, J = 9.3 Hz, 2H), 7.68 (d, J = 7.8 Hz, 2H), 7.56 – 7.42 (m, 4H), 7.38 (d, J = 7.8 Hz, 2H), 5.16 (s, 2H), 3.36 (s, 12H), 1.29 (s, 12H).; ^{13}C NMR (101 MHz, DMSO-d_6) δ 154.28, 139.29, 138.26, 135.41, 135.04, 133.96, 127.68, 119.49, 107.25, 84.20, 69.08, 41.54, 25.13. ESI-MS calculated for $C_{30}H_{37}BN_3O_4S^+$ $[M+H]^+$, 546.5; found, 546.3. HR-MS calculated for $C_{30}H_{37}BN_3O_4S^+$ $[M+H]^+$, 546.2598; found, 546.2606.

3. Determination of the fluorescence quantum yield (Φ)

The relative fluorescence quantum yield of ONP was obtained by comparing the area under fluorescence spectra of the test samples with standard samples, where using the solution of MB (10 μM, Φ = 0.11, λ_{ex} = 640 nm) for the reference (*ref: J. Am. Chem. Soc. 2018, 140, 1767−1773; Dyes and Pigments, 2015, 119, 12-21*). The fluorescence quantum yield was calculated according to the following equation:

$$\Phi_x/\Phi_s = [A_s/A_x][n_x^2/n_s^2][D_x/D_s]s$$

Where s = standard; x = sample; A = absorbance at the excitation wavelength; n = refractive index; and D = area under the fluorescence spectra on an energy scale. Optical properties of ONP (10 μM) was examined in 10 mM PBS buffer containing 5% MeCN as a co-solvent. Methylene blue was used as a calibration standard under conditions of matched absorbance at 640 nm. All fluorescence measurements were conducted for dilute solutions with an absorbance near 0.05. The fluorescence quantum yield of **ONP** was measured as 0.0069.

4. The limit of detection (LOD) of ONP

The emission spectrum of free ONP in PBS buffer (10 mM, pH 7.40, containing 5% MeCN) was collected for 20 times to confirm the background noise σ. The probe noise can be

calculated from fluorescence signals in solution without $ONOO^-$ using the root-mean-square (σ), we took 20 data points to obtain the average value before treatment with $ONOO^-$.

$$Vx^2 = \sum(y_i - y)^2$$

Where y_i is the average value from calculation and y is the measured data point. The σ noise is calculated as

$$\sigma = \sqrt{V_x^2 / N}$$

Where N is the number of data points used for the average value. The σ noise of ONP is 50.65. According to the IUPAC definition, when the signal-to-noise ratio equals 3 (S/N=3), the signal is considered to be a true signal. Thus, the limit of detection (LOD) of ONP can be extrapolated from the linear calibration curve when the signal equals three times of the noise. The linear regression curve was then fitted according to the data in the range of $ONOO^-$ from 0 to 4 μM and obtained the slope of the curve ($1.6235*10^9$ M^{-1}). The detection limit (3σ/slope) was then determined to be 93.6 nM, which facilitate the quantitative detection of $ONOO^-$ in the complex environment (*ref: J. Anal. Toxicol. 2009, 33,129–142*).

5. The measurement of lipophilicity

Lipophilicity was presented as log $P_{o/w}$ values, which were determined by the flask-shaking method. An aliquot of a stock solution of the sample in aqueous NaCl (0.9% w/v and saturated with octanol) was added to an equal volume of octanol (saturated with 0.9% NaCl, w/v), and the mixture was shaken overnight at 60 rpm to allow partitioning at 298 K. After the sample was centrifuged at 3000 rpm for 10 min, the probe content of the organic and aqueous phases was determined by UV absorbance (254 nm). Log P was defined as the logarithmic ratio of probe concentrations in the organic and aqueous phase.

6. HPLC analysis

HPLC analysis of ONP after incubation without or with $ONOO^-$ at 310 K was performed on an AGILENT 1200 system (AGILENT Co. Ltd., US). The reaction mixture was analyzed with the detection wavelength at 665 nm (for the product of MB) and 254 nm (for ONP), respectively. Running conditions were as follows: mobile phase composition was MeCN/H_2O: 65/35 (0.1% TFA); temperature of 303 K; Agilent RP-C8 column of 4.6*180mm; and flow rate of 1 mL/min.

7. Cell culture

Live SH-SY5Y human neuroblastoma cells were cultured in Dulbecco's Modified Eagle's medium (DMEM, PAN Biotech, Germany) supplemented with 10% fetal bovine serum (Life Technologies, USA), sodium pyruvate, non-essential amino acids and 100 U/mL penicillin/100 µg/mL streptomycin, and grown at 37 °C in a 5 % CO_2 incubator.

8. Cytotoxicity assay

The cytotoxic effect of ONP and MB were evaluated by employing Cell Counting Kit-8 assay (APE x BIO). Cells in the logarithmic phase were harvested and plated into 96-well plates (5000 cells/well). After 12 hours, cells were treated with different concentrations (0, 5, 10, 25, 50, 75, 100, 150 µM) of compounds. After incubation of 48 h, cells were washed by PBS and incubated in the 100 µL fresh medium with 10 µL CCK8 dye for more 2 h. After 10 min shaking on shaking table, plates were read in Infinite® M200 Pro Multimode Microplate Reader (Tecan, Switzerland) at the wavelength of 450 nm (reference wavelength 650 nm). Five replicate wells were used for each concentration. The values IC_{50} were calculated with Prism 7.0 (GraphPad Software, USA). The IC_{50} was defined as the compound concentration required to inhibit cell viability by 50%, in comparison with cell treated with the maximum amount of CH_3CN (1%) and considered as 100% viability.

9. Fluorescent imaging studies

All the imaging experiments were performed in live SH-SY5Y cells. Cells were pretreated with or without SIN-1 (100 µM), FeTMpyp (50 µM), NOC-18 (1 mM) for 1 h to induce the dynamic changes of cellular $ONOO^-$, then cells were washed by PBS and incubated with ONP (10 µM, containing 1% MeCN) and Hoechst33342 (blue, 5 µg/mL) in the fresh medium for another 30 min. In contrast, live SH-SY5Y cells were pre-incubated with or without H_2O_2 (500 µM, 1 h), LPS (1 µg/mL, 12 h), NAC (1 mM, 4 h), Aminoguanidine (500 µM, 0.5 h), then cells were imaged after incubation with ONP (10 µM) for another 30 min in the fresh medium. The microscopic imaging was used a confocal fluorescent microscope (Leica TCS SP8 MP) with an excitation filter of 633 nm and the collection wavelength range is from 650-750 nm.

10. Screening by high-content analysis

Live SH-SY5Y cells were pretreated with various anticancer agents (20 µM) or different antioxidants (20 µM) for 1 h, cells were then incubated with ONP (10 µM) for another 30 min. High-content analysis (HCA) was performed after washing with PBS three times. Images and quantitative analysis were used an HCA with an excitation filter of 630 nm and the collection wavelength range is from 680-730 nm.

11. *In vivo* NIR fluorescence imaging with ONP

5-weeks-old BALB/c nude mice were performed with intraperitoneal (*i.p.*) injection of various agents including SIN-1, Rhein, curcumin, and 3, 4-dihydroxybenzaldehyde (60 mg/kg) to induce the changes of endogenous $ONOO^-$. Images were then captured at 5, 15, 30, 45, and 60 min after intravenous (*i.v.*) injection of ONP (containing 0.5% MeCN) using IVIS

Spectrum imaging system (Nanjing University) with an excitation filter of 640 nm and the collection wavelength range is from 650-750 nm.

Kainate (KA)-induced BALB/c mice, a widely used epilepsy mouse model, were performed with intraperitoneal (*i.p.*) injection of KA (6 mg/kg). Pre-treatment with antioxidant curcumin (60 mg/Kg, 3 days) or post-treatment with curcumin (60 mg/Kg, 12 h), mice were injected with KA (6 mg/kg) via intraperitoneal (*i.p.*) injection, and then ONP (1.5 mg/Kg) were injected by intravenous (*i.v.*) injection. In vivo and Ex vivo fluorescence images of relative $ONOO^-$ levels in mice brains 60 min post-injection of ONP were further performed by using the IVIS Spectrum imaging system (Nanjing University) with an excitation filter of 640 nm and the collection wavelength range is from 650-750 nm.

Brain slices were prepared after isolating the above mice by using a vibrating blade microtome in 25 mM PBS (pH 7.4). Slices were then observed and imaged at the different depths by using a two-photon confocal fluorescent microscope (Leica TCS SP8 MP, Nanjing University) after staining with Hoechst33342 and washing with PBS. The fluorescence emission was collected at between 650-750 nm and 410-525 nm upon excitation at 633 nm and 405 nm, respectively.

12. Frozen sectioning

For the preparation of brain sections, mice were deeply anesthetized with isoflurane and sacrificed. The fresh brain was taken out and directly frozen at liquid nitrogen. Optimal Cutting Temperature (O.C.T.) Compound embedded and freezing microtome (Leica CM1950) sliced were performed. The thickness of each slice was about 10 μM. The slices were stored at -80 ºC after drying. The sections were taken out and restored to room temperature, and then stained with DAPI for 10 minutes before the fluorescent images were recorded by a confocal fluorescent microscope (Leica TCS SP8 MP). Blue channel: nuclear

fluorescence (λ_{ex} = 405 nm, λ_{em} = 410-525 nm); Red channel: ONP fluorescence (λ_{ex} = 633 nm, λ_{em} = 650-750 nm).

13. Paraffin section and HE staining imaging

Brain tissues stored at - 80 ºC were removed directly and post-fixed in freshly prepared 4% polyformaldehyde for 48 hours. For each animal, 0.4 cm in front and back of the largest hippocampus in the coronal section were cut and placed in disposable plastic embedding frame. The plastic embedding frame containing tissue was sequentially dehydrated in different concentrations of gradient ethanol. Tissue was first immersed in a mixture of pure alcohol and transparent agent for 1-2 hours, then transferred to a pure transparent agent for immersion. The plastic package frame was placed in a container containing molten paraffin in the embedding machine (JB-P5). The melted paraffin was poured into the stainless-steel encapsulation frame, the plastic encapsulation frame was opened, and the wax-impregnated tissue material blocks were cut face down into the stainless-steel encapsulation frame with warming tweezers. The wax liquid surface solidified rapidly and then became a wax block containing tissue blocks after complete solidification. Slice a wax tape of 4 μM on the slicer (RM2016) and gently hold it on the paper with a brush. Using ophthalmic tweezers, the wax band is gently spread on the water surface of 40-45 ºC. After the slices are fully flattened on the water surface of constant temperature, the wax sheet is taken into the middle of the slide and the residual water on the slide is dumped.

Remove the paraffin from the dissolved tissue gap by baking tablets in a 60 ºC oven (GFL-230) for 20-30 minutes. The slices were put into xylene I for 20 min, xylene II for 20 min, absolute ethanol I for 5 min, absolute ethanol II for 5 min, 75% ethanol for 5 min, and washed in tap water in turn. Slices were stained with hematoxylin staining solution for 3-5 minutes, washed in running tap water for 1 min, differentiated in differentiation solution, washed in running tap water for 5 min. Then slices were dehydrated with 85% and 95% gradient alcohol

for 5 min and stained with Eosin staining solution for 5 minutes. Slices were put into absolute ethanol I 5 min, absolute ethanol II 5 min, absolute ethanol III 5 min, Xylene I 5 min, Xylene II 5 min for transparent. The sections were sealed with a neutral mounting medium. Microscopic examination and fluorescence imaging were further performed by a Nikon Eclipse E100 and a Pannoramic Midi Slide Scanner, respectively. HE channel: bright field; Blue channel: DAPI fluorescence; Red channel: O fluorescence.

Neuronal death and fluorescence intensity in the CA1, CA3, and DG sub-regions were detected by fluorescent imaging and HE staining. The positive pyramidal cells of the CA1, CA3, and DG sub-regions in the hippocampus were counted with Image J software in a 250*100 μm square of each section per animal. The average of the positive cells was expressed as the relative value of the control in each subregion. All quantitative data were performed using image analysis of ZEN or Image J.

14. Supplemental Figures

Table S1. A comparison of ONP with the reported $ONOO^-$ probes.

Probe	Buffer	Λ_{ex} / λ_{em} (nm)	ε_{max} ($10^4 cm^{-1} mol^{-1}$)	Φ	LOD	NIR	In Cell	*in Vivo*	Brain	Epilepsy	HTS	Ref
**BOD-Se**	PBS/5% CH_3CN (pH 7.4)	594/680	8.9	0.96	5 μM	Yes	Yes	No	No	No	No	**1**
**Cy-NTe**	PBS/10% CH_3CN (pH 7.4)	793/820	9.539	0.0032	0.917 μM	Yes	Yes	Yes	No	No	No	**2**
**Cy-PSe**	PBS (pH 7.4)	758/775	20.37	-	-	Yes	Yes	No	No	No	No	**3**
**BDP-NGM**	PBS/EtOH, 7:3 v/v (pH 7.4)	500/512	-	0.0052	0.4 μM	No	Yes	No	No	No	No	**4**
**P2**	PBS/10% CH_3CN (pH 7.4)	480/580	-	-	35 nM	No	Yes	No	No	No	No	**5**
**ABT**	PBS/40% EtOH (pH 7.4)	405/483	-	-	26.3 nM	No	Yes	No	No	No	No	**6**
**HKYellow**	PBS (pH 7.4)	545/570	6.0	0.034	-	No	Yes	No	No	No	No	**7**
**TPHQ**	PBS/EtOH, 9:1 v/v (pH 7.4)	577/630	-	-	16 nM	Yes	Yes	No	No	No	No	**8**
**CS-ONOO**	PBS/1% DMSO (pH 7.4)	640/700	-	0.07	59 nM	Yes	Yes	Yes	No	No	No	**9**
**probe3**	PBS/52wt% MeOH (pH 8.2)	400/461	-	-	-	No	Yes	No	No	No	No	**10**
**3-HF-OMe**	Tris-HCl (pH 7.4)	365/530	-	-	65.5 nM	No	No	No	Yes	No	No	**11**
**NP3**	PBS (pH 7.4)	375/470	1.631	-	-	No	Yes	Yes	Yes	No	No	**12**
**ONP**	**PBS/5% CH_3CN (pH 7.4)**	**640/692**	**2.916**	**0.0069**	**93.6 nM**	**Yes**	**Yes**	**Yes**	**Yes**	**Yes**	**Yes**	**This work**

HTS: High-throughput screening; LOD: Limit of Detection

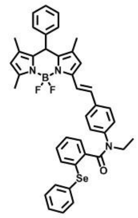
(1) BOD-Se

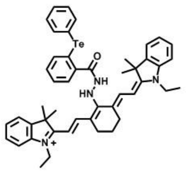
(2) Cy-NTe

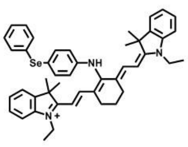
(3) Cy-PSe

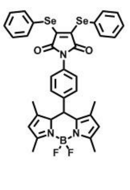
(4) BDP-NGM

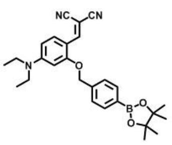
(5) P2

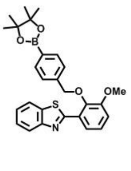
(6) ABT

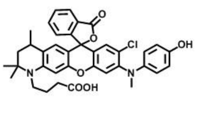
(7) HKYellow

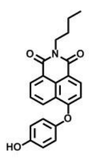
(8) TPHQ

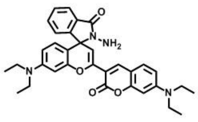
(9) CS-ONOO

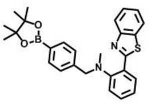
(10) probe3

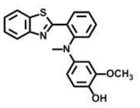
(11) 3-HF-OMe

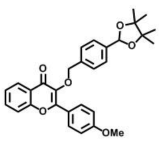
(12) NP3

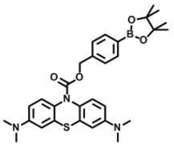
ONP

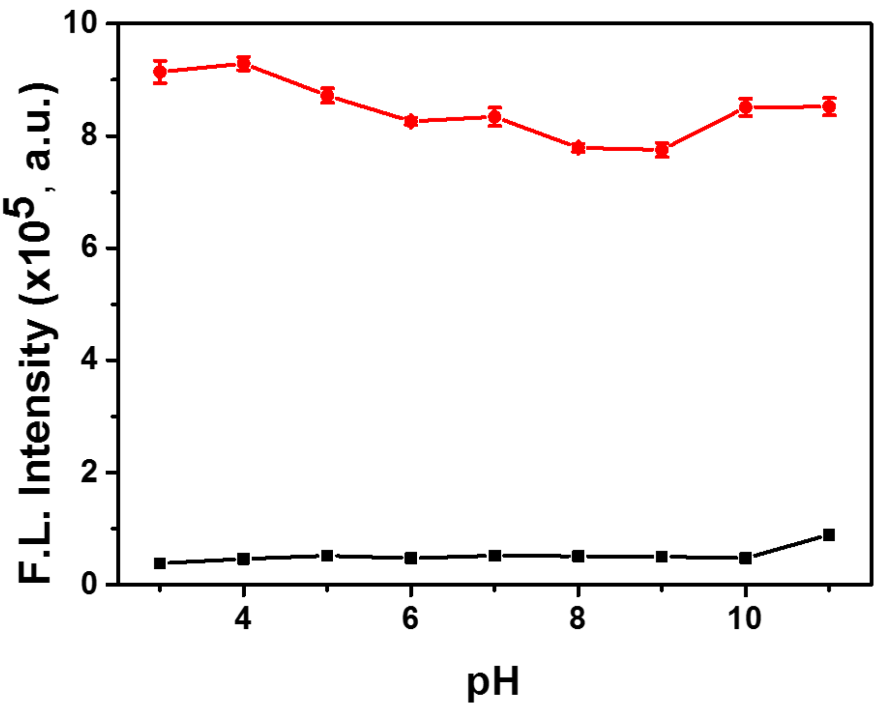

Figure S1. Fluorescence intensity changes of ONP (10 μM) towards $ONOO^{-}$ (100 μM) in PBS buffers with 5% MeCN under different pH conditions (3.0, 4.0, 5.0, 6.0, 7.0, 8.0, 9.0, 10.0, 11.0,). λ_{ex} /λ_{em} = 640/692 nm.

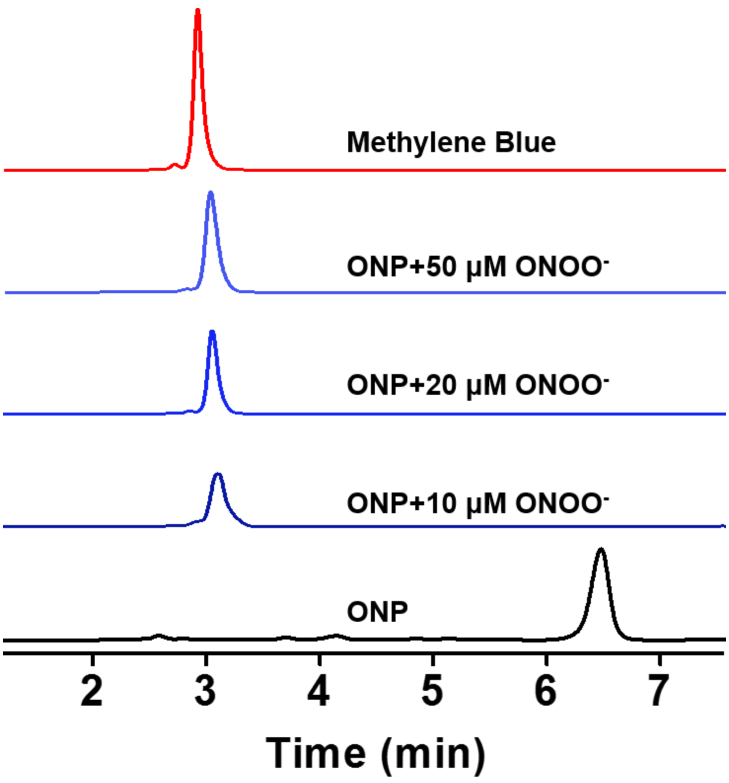

Figure S2. HPLC analysis of the reaction solution from the incubated mixture of ONP (10 μM) with various concentrations of $ONOO^-$ for 30 min in fluorescence channel (665 nm) and ONP channel (254 nm).

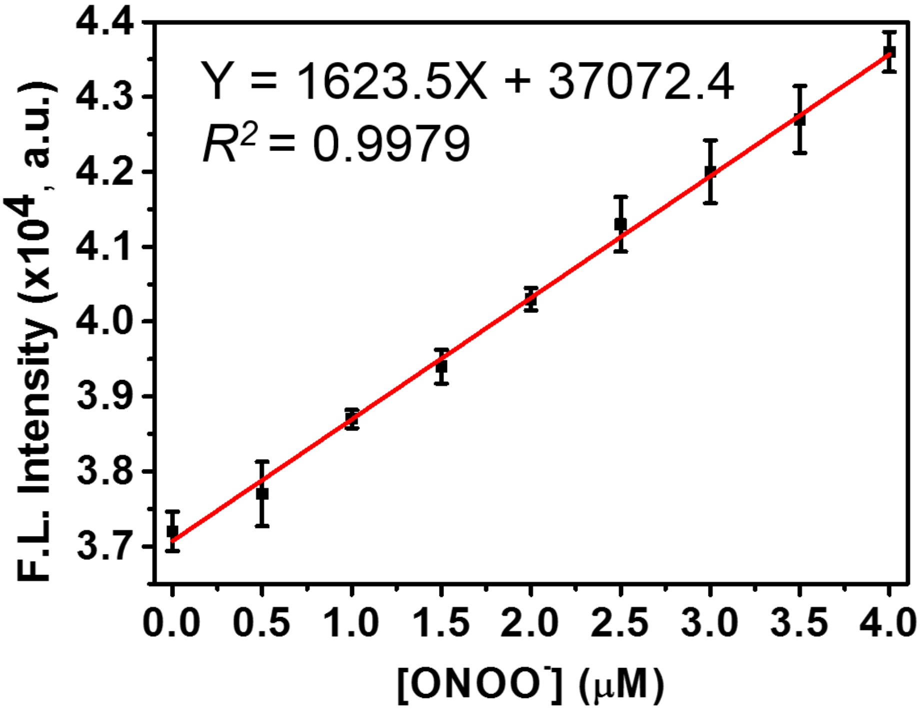

Figure S3. The linear correlation between fluorescence emission intensity of ONP and $ONOO^-$ concentration (0−4 μM). ONP (10 μM) was incubated with $ONOO^-$ in PBS buffer (pH 7.4, co-solvent 5% MeCN) at 310 K for 15 min. $\lambda_{ex}/\lambda_{em}$ = 640/692 nm, slit widths = 2.5/2.5 nm.

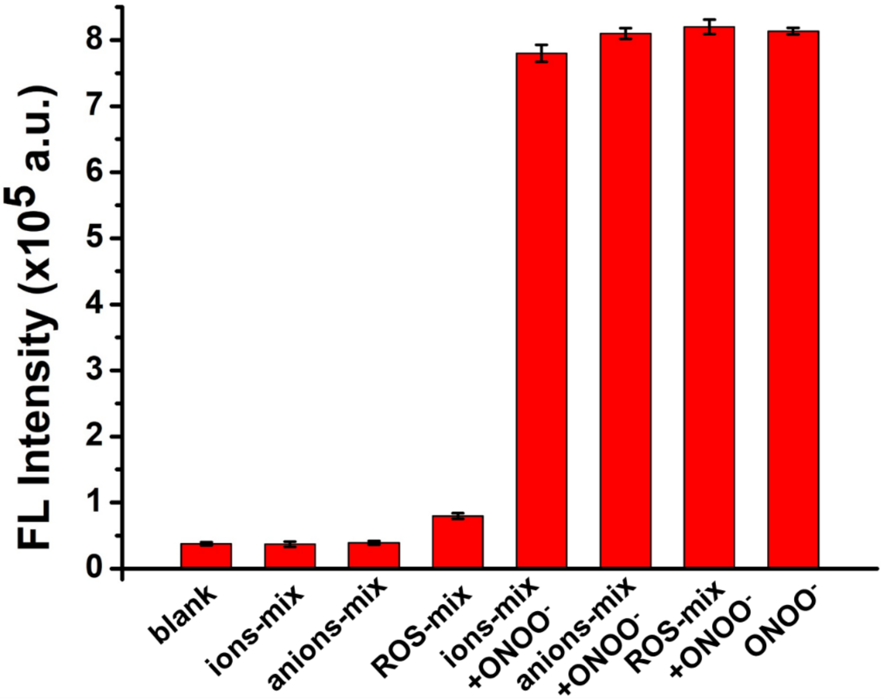

Figure S4. Fluorescence intensity of ONP (10 μM) at 692 nm after addition of 100 μM various species: (1) none, (2) ion-mix (Na^+, K^+, Cu^{2+}, Fe^{2+}, Fe^{3+} , NH_4^+, Zn^{2+}), (3) anion-mix (HS^-, SO_4^{2-}, SO_3^{2-}, NO_2^-, NO_3^-, CO_3^{2-}, PO_4^{3-}, Cl^-, ClO^-), (4) ions-mix and $ONOO^-$, (5) anions-mix and $ONOO^-$, (6) $ONOO^-$. All experiments were done in phosphate buffer (10 mM, pH 7.4, 5% MeCN) at 37 °C for 15 min with excitation at 640 nm, the data represents the average of three independent experiments.

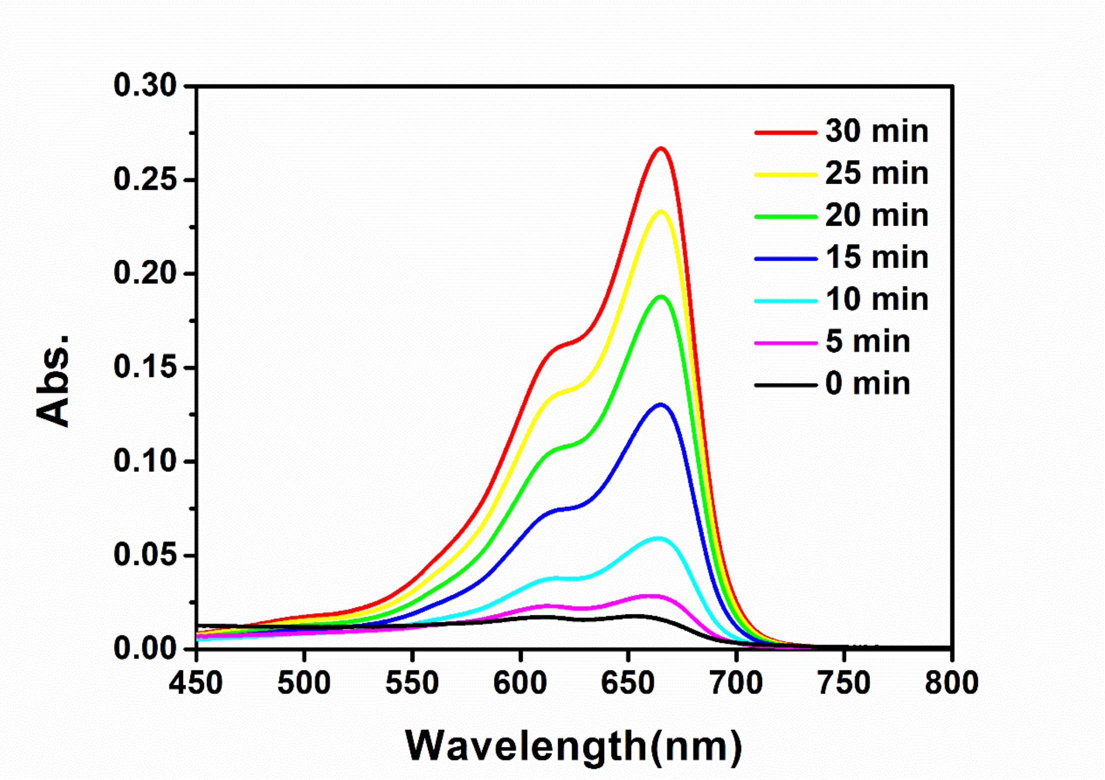

Figure S5. UV-Vis spectra of the ONP (10 μM) in PBS buffer (pH 7.4, co-solvent 5% MeCN) towards 100 μM $ONOO^-$ at 310 K for different incubation time.

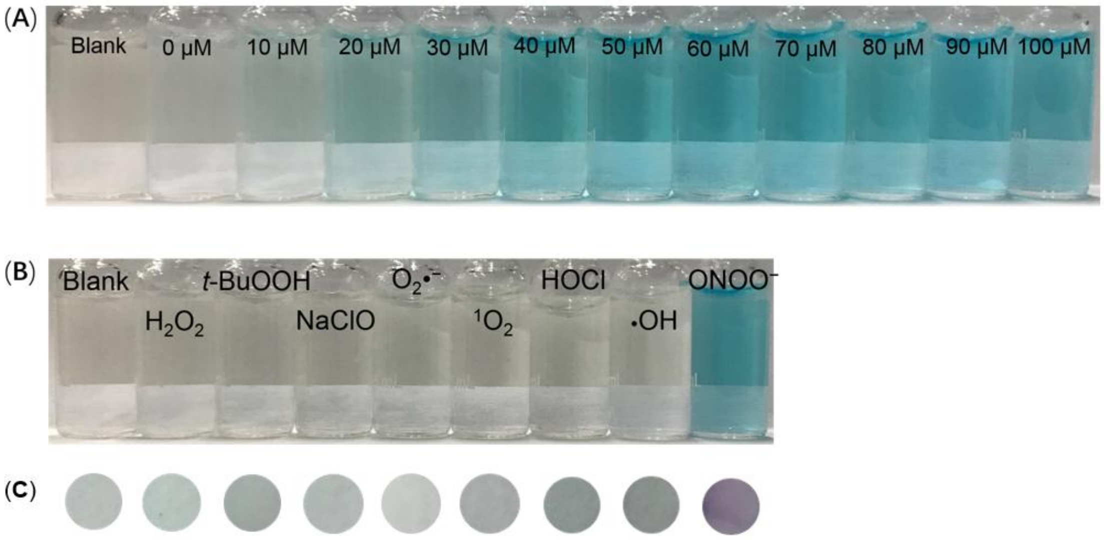

Figure S6. (A) ONP (10 μM) was incubated with different concentrations of $ONOO^-$ (0, 10, 20, 30, 40, 50, 60, 70, 80, 90 and 100 μM) at 310 K for 15 min. (B) ONP (10 μM) was incubated with various species of ROS (100 μM) at 310 K for 15 min. (C) Color changes of test strips containing ONP (200 μM) treated with various species of ROS (1 mM) at 310 K for 15 min. The photographs were taken under visible light.

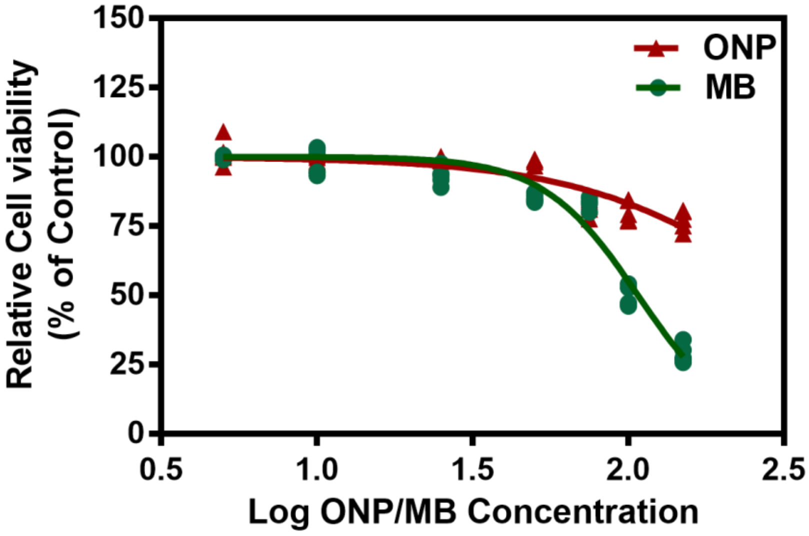

Figure S7. Cell viability of SH-SY5Y cells after treatment with different concentrations of ONP and MB. Cell viability was tested by CCK-8 assay after 48 hours of incubation. IC_{50} of ONP and MB were 340.8 µM and 107.4 µM, respectively.

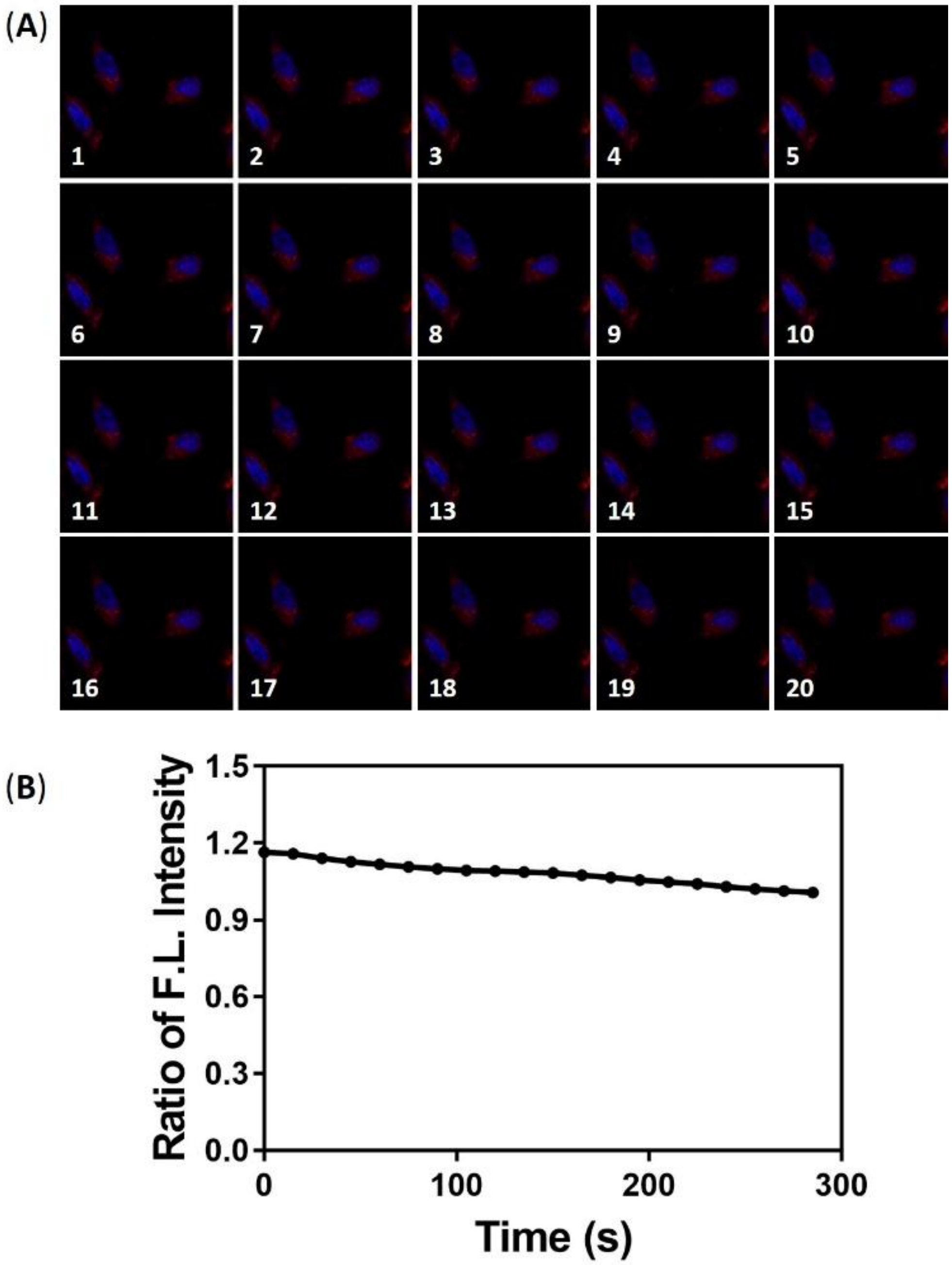

Figure S8. (A) Fluorescent images of ONP in SH-SY5Y cells under different exposure time within 285s. (B) Changes of the relative fluorescence intensity ratio *versus* different exposure time. Cells were pretreated with ONP (10 μM), subsequently, cells were imaged every 15 seconds within 285 seconds with an excitation at 633 nm. The fluorescence images of the red channel collected at 650-750 nm, excitation energy: 93.5 mW.

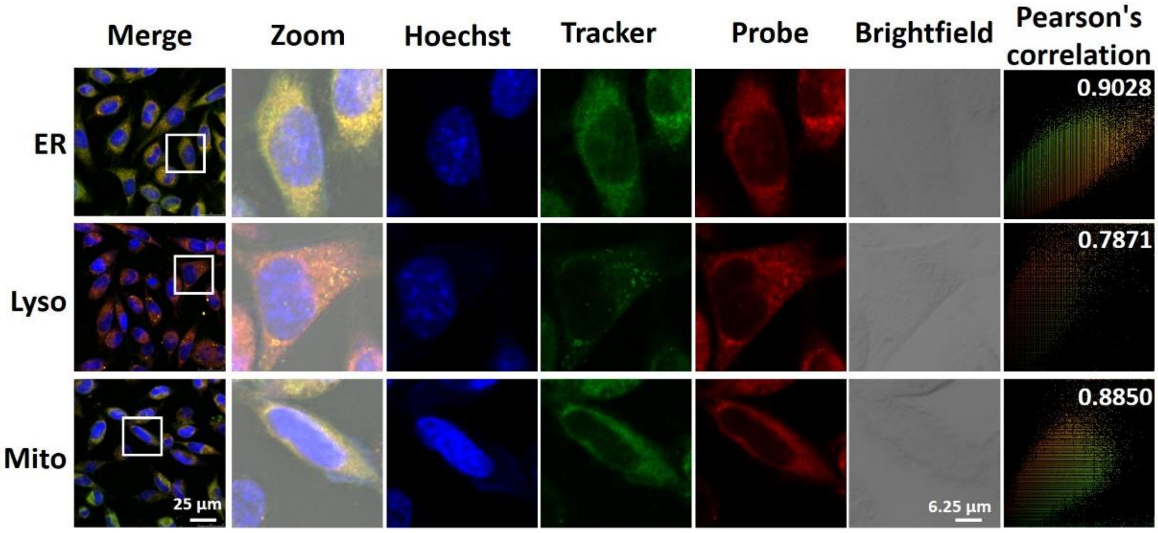

Figure S9. Intracellular co-localization of ONP in SH-SY5Y cells. Live cells were pretreated with 100 µM SIN-1 for 1 h, then incubated with ONP (10 µM) for another 30 min after washing with PBS*3 times, following Mito-, ER- and Lyso-Trackers (GREEN) were incubated before the fluorescent images were recorded by a confocal fluorescent microscope (Leica TCS SP8 MP). Blue channel: nuclear fluorescence (λ_{ex} = 405 nm, λ_{em} = 410-525 nm); Green channel: ER, Lyso, Mito-Tracker Green fluorescence (λ_{ex} = 488 nm, λ_{em} = 500-535 nm); Red channel: ONP fluorescence (λ_{ex} = 633 nm, λ_{em} = 650-750 nm). Scale bar = 25 µm; enlarged image scale bar = 6.25 µm.

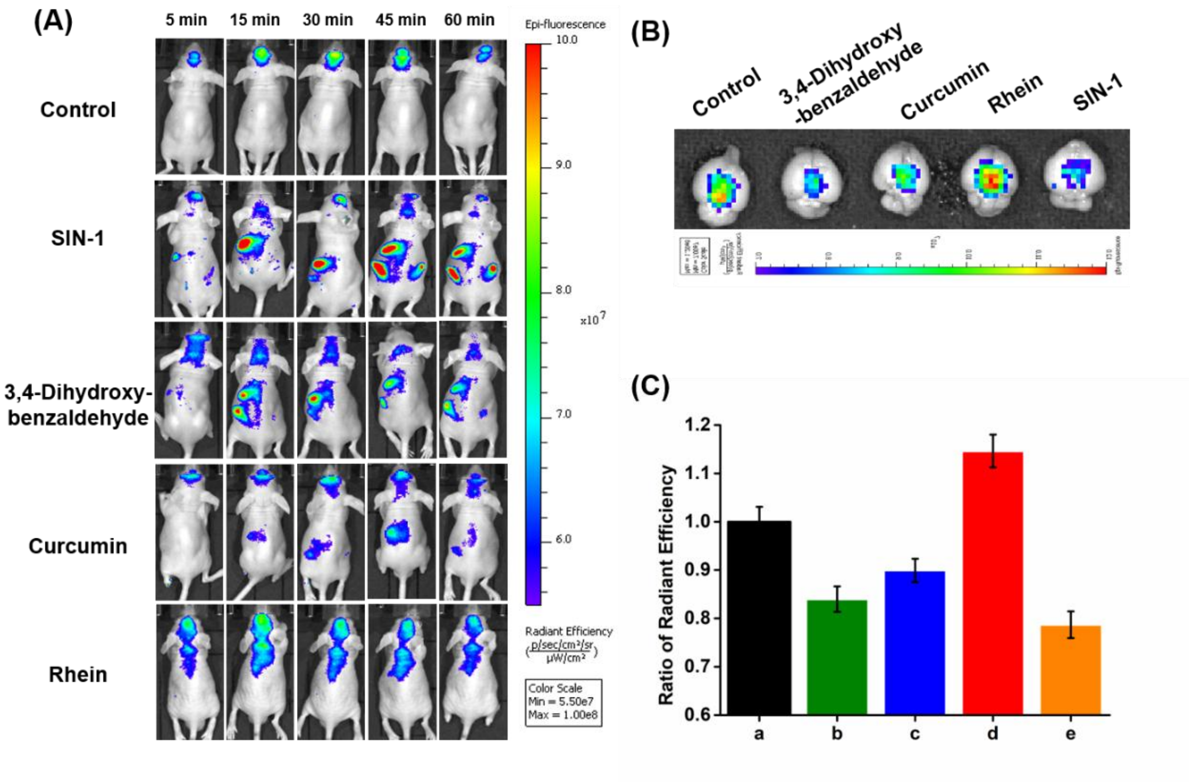

Figure S10. Mapping $ONOO^-$ Fluxes in live mice with ONP. (A) 5-weeks-old BALB/c nude mice were performed with intraperitoneal (*i.p.*) injection of different agents including SIN-1, Rhein, curcumin, and 3, 4-dihydroxybenzaldehyde (60 mg/kg) to induce the changes of endogenous $ONOO^-$. Images were captured after *i.v.* injection with ONP at 5, 15, 30, 45, and 60 min, indicating that ONP was able to cross BBB and label brains. (B) Ex vivo fluorescence images of relative $ONOO^-$ levels in mice brains 60 min post-injection of ONP. (C) The relative ratio of fluorescence intensity of the brains shown in (B) was quantified using the IVIS Spectrum imaging system.

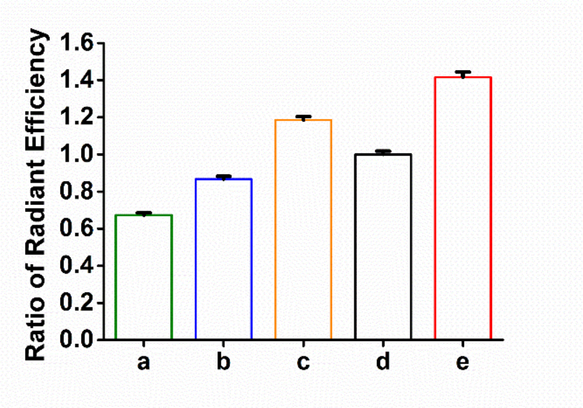

Figure S11. The relative ratio of fluorescence intensity of ex vivo fluorescence images of the brains shown in Figure 4C was quantified using the IVIS Spectrum imaging system.

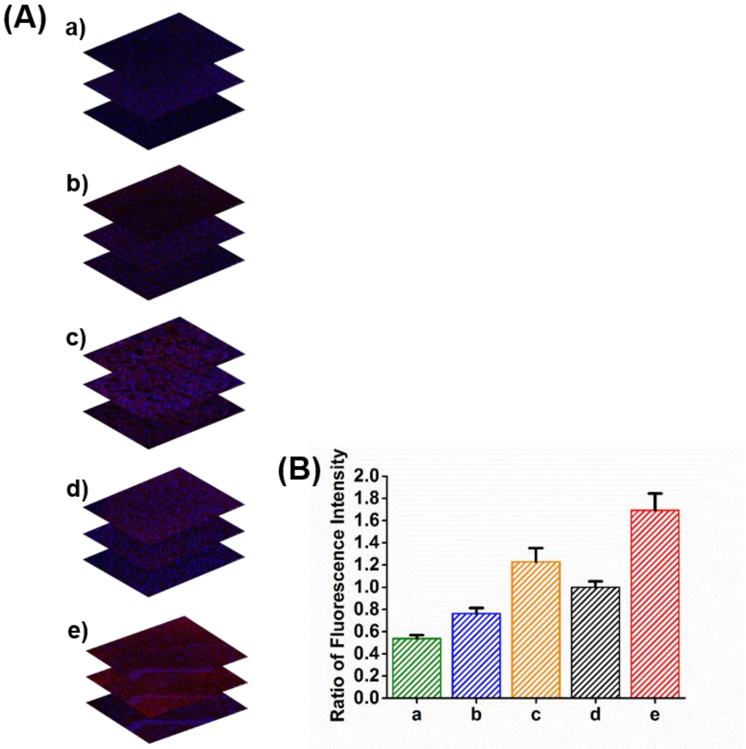

Figure S12. NIR fluorescence imaging relative $ONOO^-$ levels in mice brains 60 min post-injection of ONP. (A) Fluorescence images of brain Slices from Figure 4C. Red = ONP channel; Blue = Hoechst33342 channel. (B) The relative ratio of fluorescence intensity of the brain sections shown in (A) was quantified by Image J.

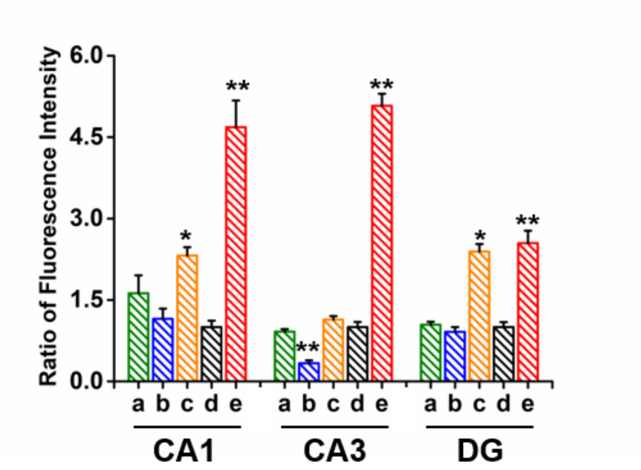

Figure S13. Quantitative analysis of ONP fluorescence in hippocampal subregions shown in Figure 4D. All ex vivo brain slices were collected from mice after 60 min post-injection of ONP, and fluorescent imaging of brain slices was performed for evaluating $ONOO^-$ concentration in the CA1, CA3, and dentate gyrus (DG) subregions of the hippocampus after KA administration. Data are presented as mean ± SD. *p < 0.05; **p < 0.01.

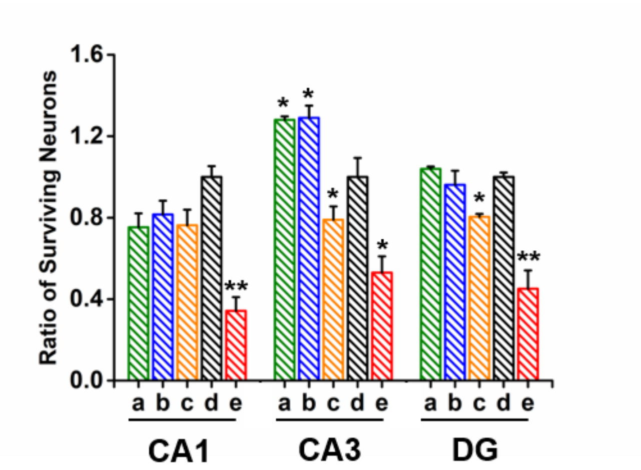

Figure S14. Quantification was performed by counting the number of positive neurons in CA1, CA3, and DG subregions of the hippocampus after KA administration. Data are presented as mean ± SD. *p < 0.05; **p < 0.01.

15. NMR and MS Spectra

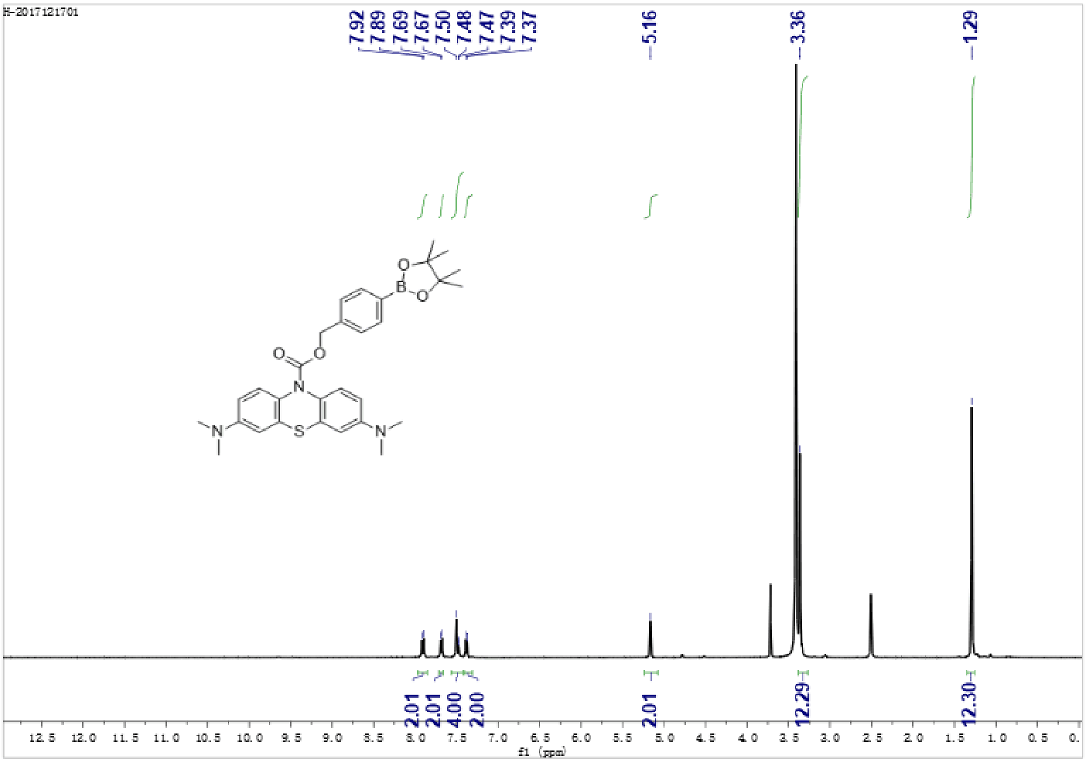

Figure S15. ^{1}H NMR spectra of ONP (DMSO-d_6).

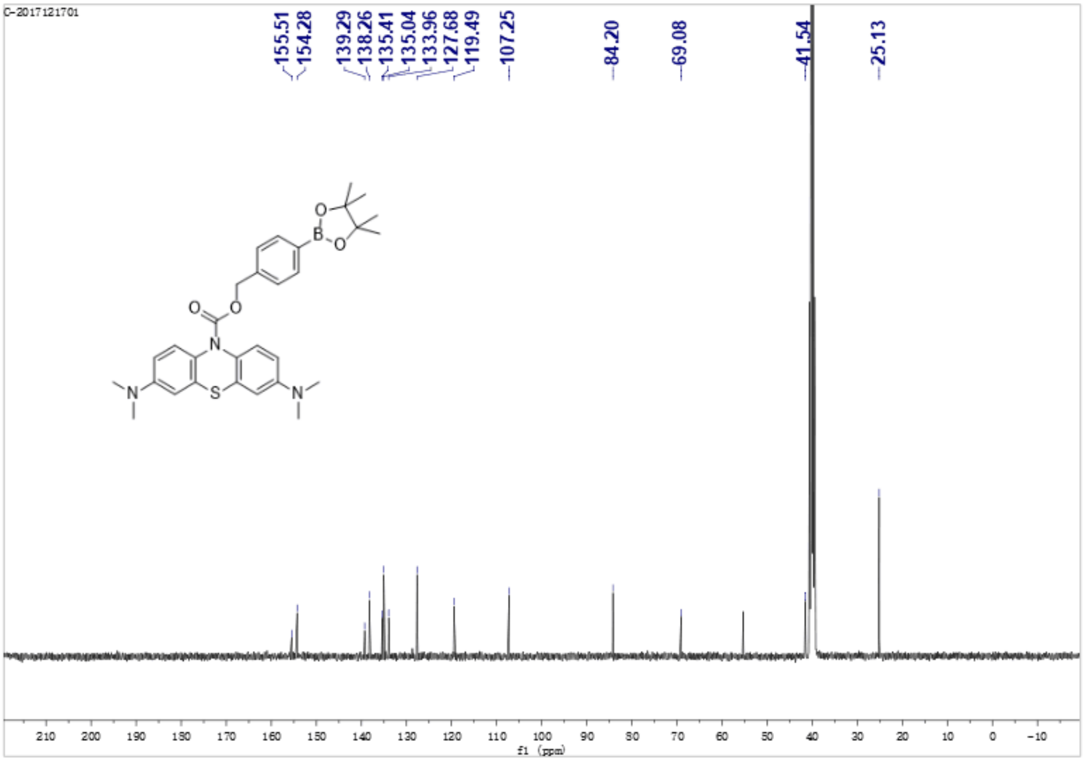

Figure S16. ^{13}C NMR spectra of ONP (DMSO-d_6).

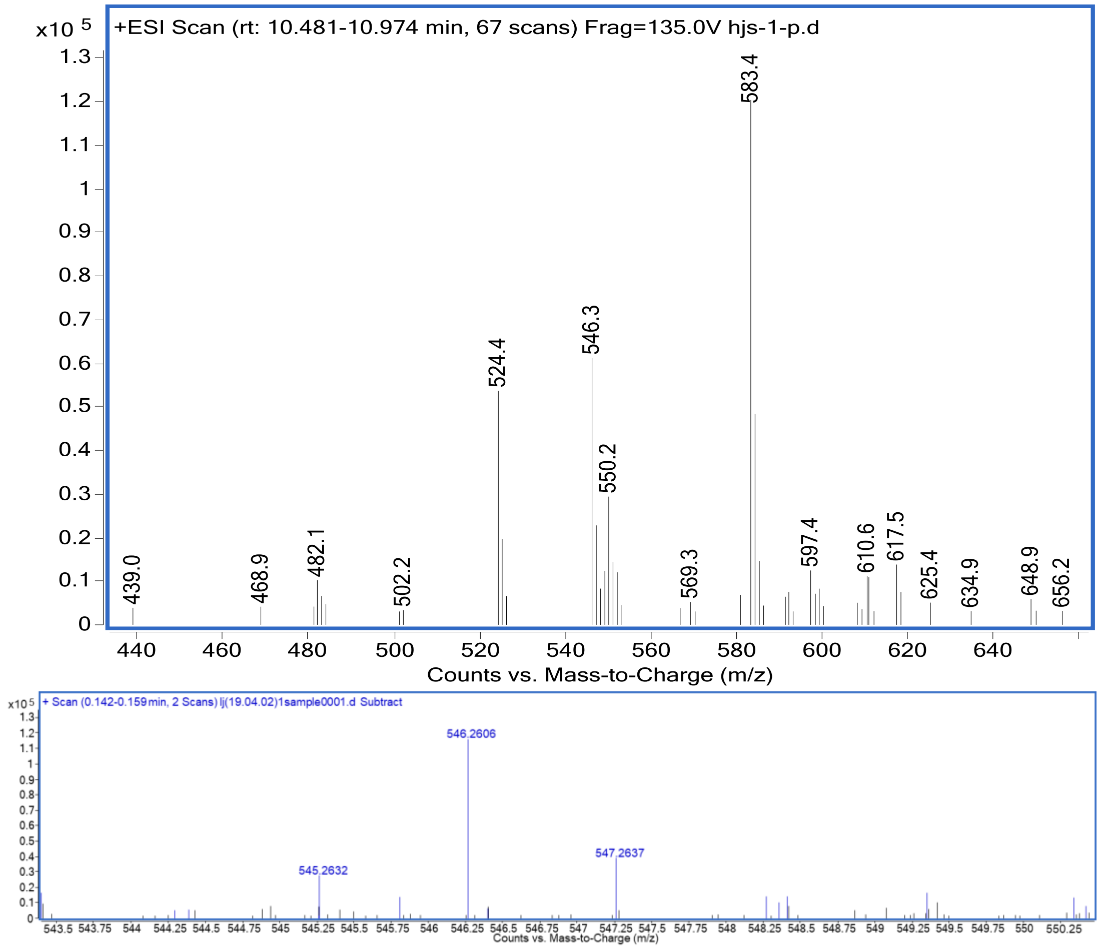

Figure S17. ESI-MS of ONP calculated for $C_{30}H_{37}BN_3O_4S^+$ $[M+H]^+$, 546.5; found, 546.3. The HR-MS of ONP calculated for $C_{30}H_{37}BN_3O_4S^+$ $[M+H]^+$, 546.2598; found, 546.2606.

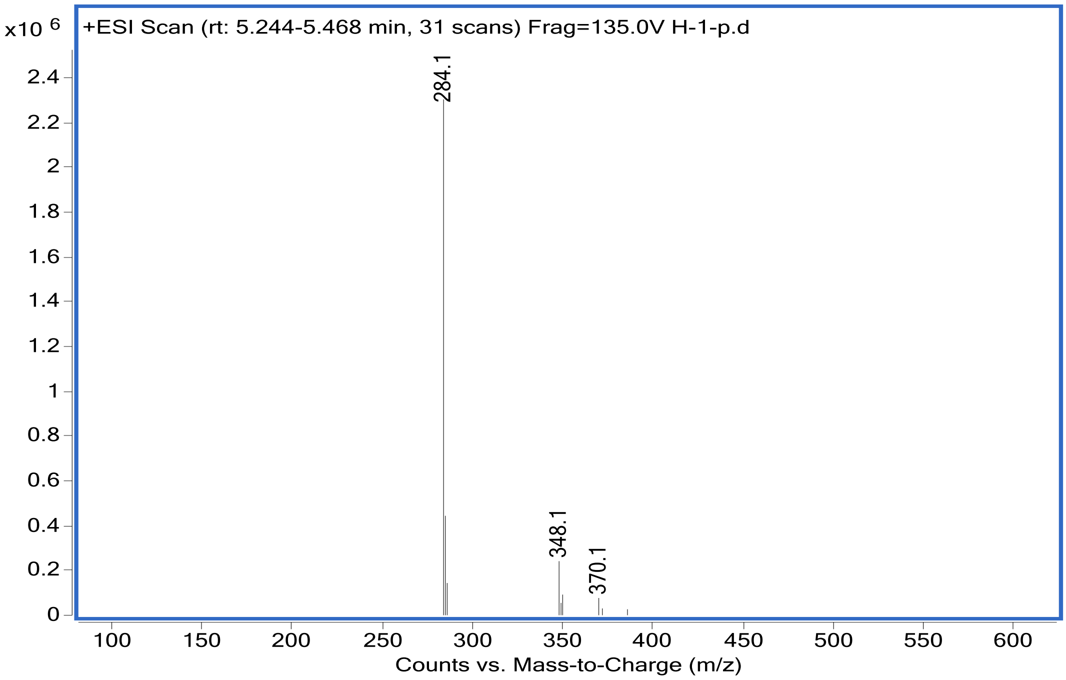

Figure S18. ESI MS of the reaction product of ONP after treatment with $ONOO^-$.

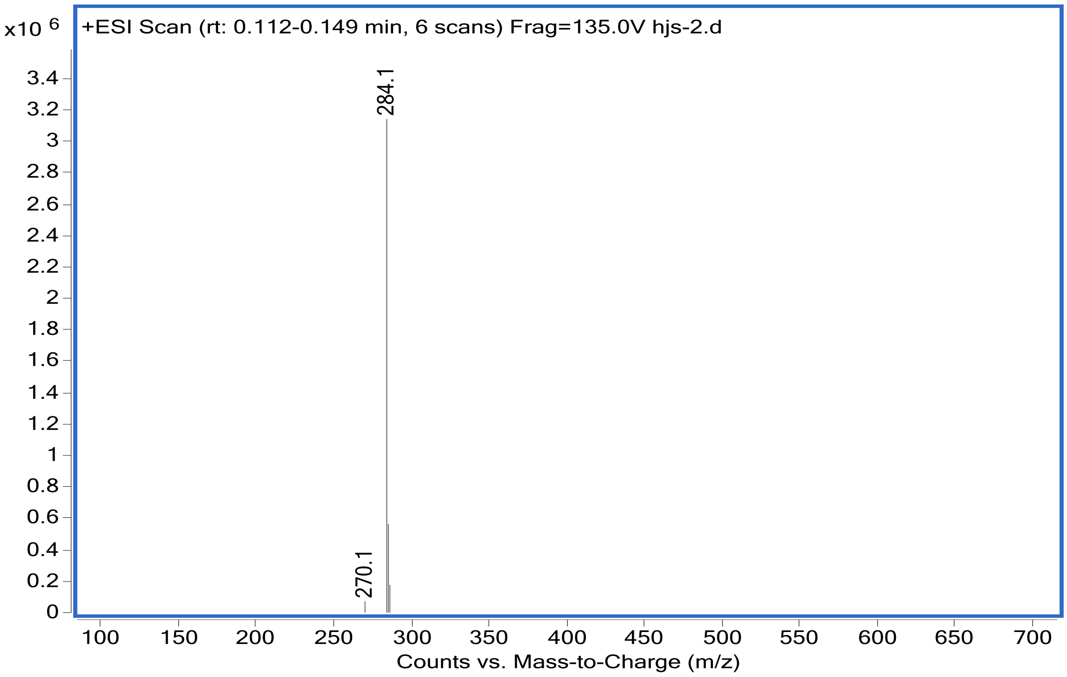

Figure S19. ESI MS of the standard MB as reference.

16. Reference

[1] B. Wang, F. Yu, P. Li, X. Sun, K. Han, *Dyes Pigments* **2013**, 96, 383.

[2] F. Yu, P. Li, B. Wang, K. Han, *J. Am. Chem. Soc.* **2013**, 135, 7674.

[3] F. B. Yu, P. Li, G. Y. Li, G. J. Zhao, T. S. Chu, K. l. Han, *J. Am. Chem. Soc.* **2011**, 133, 11030.

[4] T. Yudhistira, S. V. Mulay, K. J. Lee, Y. Kim, H. S. Park, D. G. Churchill, *Chem. Asian. J.* **2017**, 12, 1927.

[5] J. Zhou, Y. Li, J. Shen, Q. Li, R. Wang, Y. Xu, X. Qian, *Rsc. Adv.* **2014**, 4, 51589.

[6] Q. Li, Z. Yang, *Tetrahedron Lett.* **2018**, 59, 125.

[7] T. Peng, X. Chen, L. Gao, T. Zhang, W. Wang, J. Shen, D. Yang, *Chem. Sci.* **2016**, 7, 5407.

[8] B. Zhu, Z. Wang, Z. Zhao, W. Shu, M. Zhang, L. Wu, C. Liu, Q. Duan, P. Jia, *Sensor. Actuat. B-Chem.* **2018**, 262, 380.

[9] S. Feng, D. Liu, G. Feng, *Anal. Chim. Acta.* **2019**, 1054, 137.

[10] A. C. Sedgwick, X. Sun, G. Kim, J. Yoon, S. D. Bull, T. D. James, *Chem. Commun.* **2016**, 52, 12350.

[11] A. C. Sedgwick, W.T. Dou, J.B. Jiao, L. Wu, G. T. Williams, A. T. A. Jenkins, S. D. Bull, J. L. Sessler, X.P. He, T. D. James, *J. Am. Chem. Soc.* **2018**, 140, 14267.

[12] X. Li, R. R. Tao, L. J. Hong, J. Cheng, Q. Jiang, Y. M. Lu, M. H. Liao, W. F. Ye, N. N. Lu, F. Han, Y. Z. Hu, Y. H. Hu, *J. Am. Chem. Soc.* **2015**, 137, 12296.
